# The Content of Biogenic Amines in Croatian Wines of Different Geographical Origins

**DOI:** 10.3390/molecules23102570

**Published:** 2018-10-09

**Authors:** Ivana Mitar, Ivica Ljubenkov, Nikolina Rohtek, Ante Prkić, Ivana Anđelić, Nenad Vuletić

**Affiliations:** 1 Department of Chemistry, Faculty of Science, University of Split, Ruđera Boškovića 33, 21000 Split, Croatia; imitar@pmfst.hr (I.M.); angel@pmfst.hr (I.A.); nenov@pmfst.hr (N.V.); 2 University Department for Forensic Sciences, University of Split, Ruđera Boškovića 33, 21000 Split, Croatia; rohtekn@yahoo.com; 3 Department of Analytical Chemistry, Faculty of Chemistry and Technology, University of Split, Ruđera Boškovića 35, 21000 Split, Croatia; prkic@ktf-split.hr

**Keywords:** Croatian wines, biogenic amines, HPLC, geographical origin

## Abstract

Samples of white and red wines produced in two different wine-growing regions, coastal (Dalmatia) and continental (Hrvatsko zagorje) of Croatia, were analysed for biogenic amines content. Biogenic amines content was determined, and its concentration levels were associated with the geographical origin of the wine. Due to its high sensitivity, HPLC method with ultraviolet detector was used, including the derivatisation step with dansyl chloride. The method was applied to detect and quantify 11 biogenic amines in 48 red and white wines. It was found that both Dalmatian red and white wines are characterised by tryptamine (0.23–1.22 mg L^−1^), putrescine (0.41–7.5 mg L^−1^) and ethanolamine (2.87–24.32 mg L^−1^). White wines from the Hrvatsko zagorje region are characterised by content of isopentylamine (0.31–1.47 mg L^−1^), putrescine (0.27–1.49 mg L^−1^) and ethanolamine (3.80–17.96 mg L^−1^). In contrast to white wines from the Hrvatsko zagorje region, in the red wines, all biogenic amines except ethylamine, were found and equally presented.

## 1. Introduction

Recently, there has been a great interest of scientists to find a way to control the global wine market. There are numerous parameters determining the quality of the wine. These parameters can be classified as chemical and sensory parameters. Wine is a beverage wherein the quality depends on many factors, among which grape variety, origin, vintage, grape growing conditions, winemaking practice and maturation process, physical conditions of production and way of storage, are the most significant, and they also influence sensory characteristics. Over the past century, chemists have played a significant role in the determination of wine chemical composition and its association with wine flavour and sensory attributes. In the global wine market, wine identity (brand, type, vintage and origin) is extremely important and all those characteristics are crucial for the determination of its price. In the past century, chemists had developed powerful tools for detecting adulteration of wine, such as the addition of water, glycerol, alcohol, dyes, sweeteners, flavour substances and a non-authorised addition of sugar or acidity adjustment [1]. Therefore, in the last few years, there has been a great interest from scientists as well as consumers and commercial wine producers, on the geographical origin and authenticity of wines in terms of quality and price determination. In the 19th and early 20th centuries, the focus was on detecting fraud, while more recently the emphasis has been on quantifying trace compounds, especially those that may be related to a grape variety [2]. There is a large number of studies dealing with the classification of wines according to geographical origin, e.g., assuming that the concentrations or ratios of some chemical parameters in wine depend on its geographical origin [1]. According to some studies, volatile components [3], polyphenols [3,4,5,6,7], as well as elemental composition [8,9,10,11], can be linked to the geographical origin or grape variety.

Biogenic amines (BAs) are in the focus of wine quality analysis, and generally of food quality control of many studies. BAs can be found in different fermented foods, such as milk, cheese or beer as well as in wine. They are low molecular weight organic compounds. In wine they can originate from grapes, or can be produced during fermentations (alcoholic and malolactic). In most wines, the level of BAs is low after alcoholic fermentation, while after malolactic fermentation their level increases [12]. Also, they can be formed during wine ageing or storage processes, especially if wine is exposed to the microorganisms’ activity or free amino acids are present in it. The formations of BAs from free amino acids can be through reaction of decarboxylation (histamine, tyramine, putrescine, cadaverine), transamination, reductive amination or degradation of their amino acids precursors [12]. The conditions such as storage temperature, pH, and presence of oxygen, sulphur dioxide or sodium chloride content in wine are important factors that affect the concentration of BAs in the final product [13,14,15,16,17,18]. All mentioned conditions act synergistically. Obviously, with some combinations, BAs may be increased through progressive fermentation process, e.g., elevated storage temperature, pH and O_2_ presence. On the other hand, elevated SO_2_ and NaCl concentrations diminish fermentation process and decrease BAs content in wines.

Although BAs are considered essential for many physiological functions, such as: body temperature regulation, stomach pH value and brain activity, the frequent and prolonged intake of BAs through wine consummation causes various health problems, such as headaches, flushing, itching, skin irritation, hypertension, etc. [13,15]. BAs are found to be toxic in cases of the intake of foods or beverages that contain them in large amounts. BAs suspected of having toxicological effects are histamine, tryptamine and phenethylamine. Therefore, the level of BAs in wine can be a safety indicator as well as an important parameter for grading wine quality [15]. Polyamines are usually associated with deficient sanitary conditions, especially putrescine and cadaverine. There is a large variability in content and distribution of BAs in wine.

A large number of studies on BAs in commercially available wines have been carried out in the world’s most important wine producing countries: Turkish red wines [19], Greek red wines [20,21], Chinese red wines [22], Chilean wines [23], Brazilian wines [16], Portuguese [24] and Spanish red and white wine samples [25,26,27], purchased from local stores with the aim of monitoring or determining the content of biogenic amines, the high content of which disrupts the quality or proves bad hygiene conditions of wine. More than 20 biogenic amines have been identified in the mentioned studies.

However, there are only a few studies on BAs content with the purpose of determining wine geographical origin or grape variety. BAs are naturally present in grapes (putrescine, spermidine, histamine and tyramine) [28] and in initial musts (ethanolamine, tyramine, putrescine, cadaverine, phenylethylamine and spermidine) [29]. Del Prete et al. detected ethanolamine, ethylamine and putrescine in grapes [30], while Ladente et al. found that differences in putrescine concentration may be attributed to certain grape varieties [31]. According to those studies, the concentrations of BAs that are naturally present in must are directly connected to a grape variety or a soil type, and finally, to the grape’s geographical origin. Marques et al., explained a correlation between BAs and grape variety on the samples of red wines produced in three different Portuguese regions [32], while a group of Italian researchers in their study on red wines concluded that BAs composition is a feature of a particular geographic region [4]. Red wines are richer in the content of biogenic amines [15], which can be related to the fact that secondary fermentation (malolactic fermentation) is less usual in white wines [21,27]. Therefore, significantly smaller number of studies are conducted on white wine samples.

Croatia, like many other Mediterranean countries such as Spain, France, Italy, Greece and Turkey, has a long-standing tradition of wine production. In Croatia, there is a large number of small manufacturers who produce small quantities of wine, so the Croatian market is also known as a market of a large number of monovarietal wines [33]. As the authors know, there are only a few studies on BAs content in Croatian wines. Kovačević Ganić et al. investigated BAs content in red wine samples from Slavonia wine region and changes in BAs content during the winemaking and maturation processes. They reported the presence of 10 BAs: tryptamine, hydroxylamine, phenylethylamine, putrescine, cadaverine, histamine, tyramine, serotonin, spermine and spermidine in investigated samples [14]. Jeromel et al. investigated the concentration of BAs in red wines from northwest Croatia with the aim of comparison of BAs levels in wines produced by classic and cold maceration. In their study, the most abundant BAs were histamine, tryptamine and 2-phenylethylamine, while tyramine, putrescine, cadaverine, spermidine, spermine and serotonin were also detected in significantly lower concentrations [34]. There is also a lack of studies on BAs in Croatian white wine samples.

Although numerous analytical methods have been reported for the determination of the BAs in wines and other beverages or food samples [24,35,36], high-performance liquid chromatography (HPLC) is preferred by most researchers [27,30,37,38,39]. There are differences in the derivatisation procedures among studies, but the most commonly used reagents are *o*-phthalalhehyde (OPA), dabsyl chloride (DABS-Cl) and dansyl chloride (DNS-Cl).

Although, researchers are usually using OPA as a derivatisation agent, we used dansyl chloride because of its stability when exposed to UV-Vis spectra (in detection system). Also, OPA reacts only with primary amines, which prevents the determination of polyamines such as spermine and spermidine whose presence has been previously reported in Croatian wines.

In this study, 48 samples of Croatian white and red wines were analysed. The investigated wines were produced from native and introduced grape varieties, characteristic of two Croatian wine regions; Dalmatia (coastal wine region) and Hrvatsko zagorje (continental wine region). Also, this research was conducted with the aim of comparison of the BAs concentration in red and white wines from the same wine regions.

## 2. Results

### 2.1. Samples

A total of 48 samples of wines from two different Croatian wine regions are listed in Table 1 (samples from coastal wine region of Dalmatia) and Table 2 (samples from continental wine region of Hrvatsko zagorje) with corresponding grape variety and origin. All wine samples were obtained directly from small farmers of the corresponding region.

### 2.2. Biogenic Amines Determinations

The content of biogenic amines was determined by HPLC method, as described by Manetta et al. [40], with slight modifications.

The derivatisation was performed without pre-treatment of the samples as follows: 0.25 mL of BAs standard solution or wine sample was mixed with 70 µL of a saturated sodium hydrogen carbonate solution and 65 µL 0.1 M potassium hydroxide solution. Then, 1 mL of dansyl chloride (0.5% *w/v* in acetone) was added and the mixture was incubated for 45 min at 40 °C in thermoblock with occasional stirring. After that, 100 µL of ammonia solution (25% *w/w*) was added and after strong stirring by vortex, the reaction mixtures were left in dark for 30 min. The volume of samples was made up to 5 mL with acetonitrile, and after the shaking they were filtrated and ready for the analysis. The control samples (blank) was prepared by the same procedure, but instead of standards or wine samples, ultrapure water was used.

All samples were prepared and analysed in triplicate, and the data are presented as a mean value ± standard deviation.

Gradient elution was conducted using acetonitrile (solvent A), and ultrapure water (solvent B) according to the program presented in Table 3.

**Figure 1:**
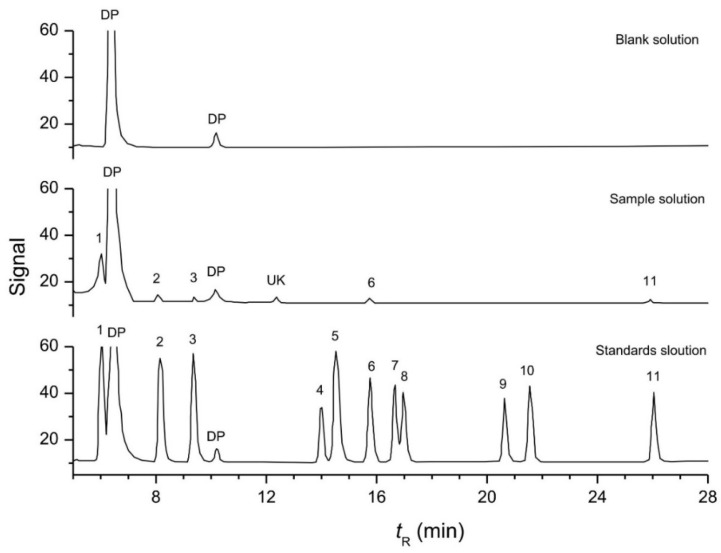
HPLC chromatogram of the blank sample, wine sample and standards’ solution of biogenic amines. The numbers correspond to amines reported in Table 4, DP—derivation peak, UK—unknown peak.

The applied flow rate was 1 mL min^−1^, column temperature was 25 °C, the sample injection volume was 10 µL and the detection wavelength was 254 nm. The identification of BAs was carried out by comparing their retention times, individually or in a mixture of standards as is shown at Figure 1. Peaks appearing in the chromatogram that are not assigned to any standard are secondary products of the derivatisation process. The quantification was done using linear calibration curves that were created for every standard compound of BAs.

As ethanolamine, ethylamine and methylamine were obtained as hydrochloride salts, their concentrations in standard solutions were corrected as for a free base.

The limits of detection (LODs) and the limits of quantification (LOQs) were determined using signal-to-noise-ratio (S/N) of 3 and 10 respectively for all standards. Table 4 is showing calculated analytical parameters for the applied method.

The detected concentrations of 11 investigated biogenic amines (tryptamine, putrescine, cadaverine, histamine, tyramine, spermidine, spermine, isopentylamine, ethanolamine, methylamine and ethylamine) in 48 samples of Croatian red and white wines from Hrvatsko zagorje and Dalmatia wine regions are given in Table 5. The samples can be divided into four groups: Dalmatian white wines (11 samples), Dalmatian red wines (13 samples), Hrvatsko zagorje white wines (10 samples) and Hrvatsko zagorje red wines (14 samples). The results are presented in Table 5 as the average value ± relative standard deviation (RSD) (%) of all samples.

## 3. Discussion

In the case of polyamines (putrescine, cadaverine, spermidine and spermine), their high concentrations are usually associated with unsanitary conditions.

If we look at the detected concentrations of those polyamines in the investigated samples, in most white wines cadaverine, spermidine and spermine were not detected, while in red wines the highest concentrations detected were 1.98 mg L^−1^ of cadaverine, 2.51 mg L^−1^ of spermidine and 3.55 mg L^−1^ of spermine. Therefore, it can be concluded that the sanitary conditions of all the samples were satisfactory.

Histamine level also plays a special role as indicator amine. Histamine is the most toxic amine, although the toxicity is caused by histamine and the total content of amines, ethanol and acetaldehyde [41]. The allowed concentrations of histamine in wines are different across countries. According to available references, the highest histamine concentration of 10 mg L^−1^ is allowed in Switzerland [42]. According to the reported results, the highest concentration of histamine among tested samples was observed in the 3ZC sample (9.63 mg L^−1^), while its content in other red wine samples was generally low (from 0.35 to 2.01 mg L^−1^), and in white wines even below the detection limit, except for sample 8 DB, where it was found at concentration of 0.56 mg L^−1^.

Putrescine and ethanolamine were the most prominent amines in all of the samples, regardless of the type of wine or its origin.

According to the Bover-Cid et al. [25], Glória et al. [43] and Kiss et al. [44] putrescine, spermine and spermidine are naturally present in grapes and their presence could be an indicator of the wine’s geographical region or grape variety.

Putrescine concentrations were slightly higher in red wines, which can be explained by the fact that it can be formed during malolactic fermentation that usually occurs in the process of red wine production [25]. The highest concentration of putrescine in red wine samples was found in sample 2DC (7.5 mg L^−1^). In comparison to results reported in other studies, these concentrations were relatively low [15,16,19,21,22,23,24,27,30,37,45,46,47]. The putrescine content in samples was investigated by Landete et al. ranged from 30 to 50 mg L^−1^ [31], while in our study they ranged from 0.16 to 3.75 mg L^−1^. Spermidine was not detected in white wines, except in the sample 8ZB where its concentration was 0.12 mg L^−1^. In red wine samples, spermidine was found in the range from 0.09 to 0.46 mg L^−1^ in Dalmatian red wines and from 0.15 to 6.05 mg L^−1^ in wine samples from Hrvatsko zagorje region.

Spermine was found in low concentrations in four samples of white wines from Dalmatian region (ranged from 0.11 to 0.39 mg L^−1^), while in white wines from Hrvatsko zagorje region it was not detected. If we compare the results for spermine in red wines, slightly higher concentrations were detected in samples from Hrvatsko zagorje region than in Dalmatian wines but final concentrations correspond to those that are reported in the literature [20,43].

Del Prete et al. confirmed the presence of ethanolamine, ethylamine and putrescine in grapes [30].

Ethanolamine is an amine that was detected in almost all samples at significant concentrations and especially in red wines from Hrvatsko zagorje region. Its concentrations correspond to those reported in other studies of Mediterranean wines, such as in samples from Italy [4,30], Portugal [47], and Greece [20]. A large number of studies didn’t research the ethanolamine content, but in our study, its concentration was found to be significant in almost all of the samples.

Ethylamine was detected only in sample 12ZC, and thus in a very low concentration of 1.17 mg L^−1^, as well as methylamine, which was found in sample 3DB and in few red wines from Hrvatsko zagorje wine region at concentration range from 0.99 to 1.57 mg L^−1^.

Jeromel et al. studied BAs in Croatian red wines, but in their study the content of ethylamine and methylamine were not investigated [34], while histamine and tryptamine were found to be the most abundant biogenic amines.

In this study, tryptamine was detected in the range from 0.2 to 1.2 mg L^−1^ in the wines from Dalmatia, while in the red samples from Hrvatsko zagorje region it was found in higher concentrations (from 0.09 to 9.18 mg L^−1^).

It is interesting to see the distribution of isopentylamine in the samples. Its concentrations in the samples from Hrvatsko zagorje ranged from 0.3 to 1.5 mg L^−1^ in white wines and from 0.33 to 3.71 mg L^−1^ in red wines, while among Dalmatian samples, only samples 2DB and 2DC contained this compound in concentration from 0.29 and 1.13 mg L^−1^, respectively.

Marques et al. in their research proved the connection between the content of tyramine and type of wine especially as its concentration is higher in red wines after malolactic fermentation [32].

Tyramine was found in very low concentrations in two white wines from Dalmatian region, while it was not detected at all in white wines from Hrvatsko zagorje region. As expected, red samples contained higher amounts of tyramine, but still significantly lower than those reported in the literature [32,45,46]. A very wide range of total BAs content has been reported, from not-detected to 130 mg L^−1^ with the main amines e.g., putrescine, histamine, tyramine and cadaverine [48].

**Figure 2:**
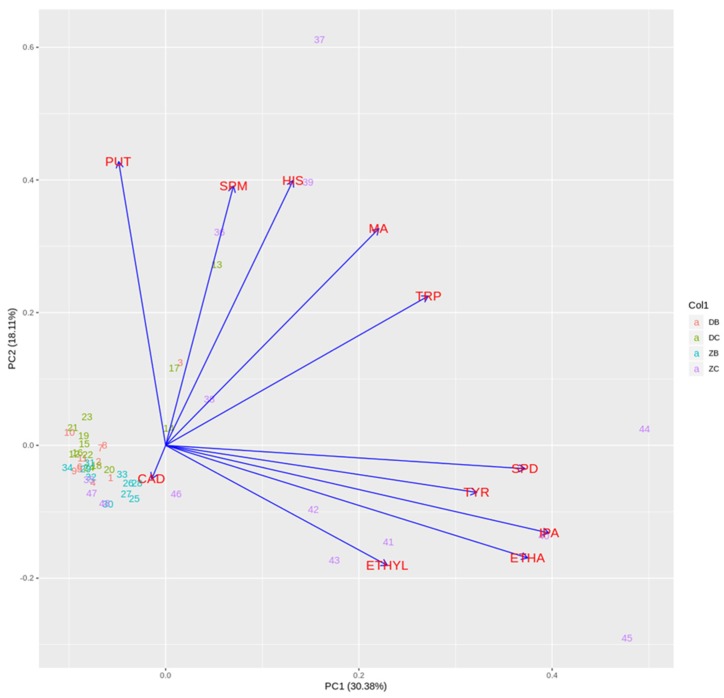
PCA plot. Table 4 contains abbreviations for biogenic amines. Numbers correspond to samples’ in Table 1 and Table 2. DB = Dalmatian white samples, DC = Dalmatian red samples, ZB = Hrvatsko zagorje white samples and ZC = Hrvatsko zagorje red samples.

A scree plot in Figure 2 suggests involving four principal components in the model. Those four PC (columns PC1–PC4) explain 70% of the total variance in the data, (Table 6). Since graphical presentation only allows for using two columns, the cut off point for loading values was >0.30 and it is marked throughout Table 6 in boldface type only for PC1 and PC2.

The loading values express how well the new PCs correlate with old variables. The first PC, which explains 30.38% of the total variance correlates positively with Tryptamine (TRP), Isopentylamine (IPA), Tyramine (TYR), Spermidine (SPD) and Ethanolamine (ETHA). The second PC (18.11% of the total variance) correlates positively with Putrescine (PUT), Histamine (HIS), Spermine (SPM) and Methylamine (MA). On the other hand, it can be seen that CAD has small values. All data are given in Table 6 and Table 7.

## 4. Materials and Methods

### 4.1. Chemical and Reagents

All used reagents were of analytical grade.

Dansyl chloride and amine standards (isopenthylamine, ethanolamine, methylamine, ethylamine, spermidine, spermine, putrescine, tyramine, histamine, cadaverine and tryptamine) were purchased from Sigma-Aldrich (Steinheim, Germany).

Hydrochloric acid (37%, *w/w*), ammonia solution (25%, *w/w*), sodium hydrogen carbonate, acetonitrile (HPLC grade) were also purchased from Sigma-Aldrich.

Ultrapure water was obtained from ELGA Purelab flex.

### 4.2. Apparatus and Software

A Perkin Elmer Series 200 HPLC system, equipped with an autosampler, binary pump and UV/Vis detector (all of Series 200), was applied with a TotalChrom Workstation software (PerkinElmer, Waltham, MA, USA).

Chromatographic separations were performed on a Restek Ultra IBD C18 column (5 µm particle size, 250 × 4.6 mm i.d.) with Ultra IBD guard column (5 µm particle size, 10 × 4 mm i.d.), Restek, Bellefonte, PA, USA.

### 4.3. Statistical Analysis

For exposing the underlying patterns in the data, principal component analysis (PCA) was used with the intention of showing which biogenic amines (wine samples) carry comparable information, and which of them are unique. The statistical analysis was carried out using the RStudio ver. 1.1.383 [49] while PCA analysis was done using ‘prcomp’ method by using singular value decomposition (SVD). Since SVD has slightly better numerical accuracy, therefore, ‘prcomp’ is the preferred function.

Biogenic amines were taken as variables (columns of the input matrix) and the various wines as cases (rows of the matrix). The underlying patterns, ‘components’ are represented by new variables called principal components.

## 5. Conclusions

This work shows that biogenic amines content can be a differentiation factor for a grape variety and geographical origin for red wines. It can be stated that Dalmatian white wines are characterised by tryptamine, putrescine and ethanolamine. Their content is in the following ranges: tryptamine from 0.23 to 1.22 mg L^−1^; putrescine from 0.41 to 7.5 mg L^−1^ and ethanolamine from 2.87 to 24.32 mg L^−1^. White wines from the Hrvatsko zagorje region are characterised by content of isopentylamine (from 0.31 to 1.47 mg L^−1^), putrescine (from 0.27 to 1.49 mg L^−1^) and ethanolamine (from 3.80 to 17.96 mg L^−1^). On the other hand, in red wines from the Hrvatsko zagorje region all BAs, except ethylamine, were found. According to the PCA, the wines of the Hrvatsko zagorje red group samples are the most distinguished. Wines from the Hrvatsko zagorje red group marked as 36, 37 and 39 have a higher concentration of spermine, histamine, methylamine and putrescine, as well as lower concentration of spermidine, tyramine, isopentylamine, ethanolamine and ethylamine than wines from the same group in lines 41–45. All other wines are mostly concentrated around similar values, with the exceptions of Dalmatian red wines marked as 13 and 17, and Dalmatian white wine marked 3, respectively.

## Figures and Tables

**Table 1:** The investigated red and white wine samples from Croatian coastal wine region of Dalmatia.

Number	Sample Mark	Grape Variety	Origin	Type of Wine
1	1DB	Debit	Drniš	white
2	2DB	Pošip	Bol, Brač	white
3	3DB	Pošip barrique	Bol, Brač	white
4	4DB	Kujundžuša	Rogoznica	white
5	5DB	Maraština	Kaštela	white
6	6DB	Maraština	Bol, Brač	white
7	7DB	Chardonnay	Kaštela	white
8	8DB	Pošip barrique	Kaštela	white
9	9DB	Kujundžuša + Graševina	Imotski	white
10	10DB	Debit	Sinj	white
11	11DB	Debit	Šibenik	white
12	1DC	Plavina + Lasin + Shiraz	Drniš	red
13	2DC	Bogondon	Bol, Brač	red
14	3DC	Tribidrag	Bol, Brač	red
15	4DC	Plavac	Rogoznica	red
16	5DC	Plavac	Kaštela	red
17	6DC	Plavac	Bol, Brač	red
18	7DC	Crljenak	Kaštela	red
19	8DC	Plavac	Kaštela	red
20	9DC	Crljenak	Bol, Brač	red
21	10DC	Crljenak	Imotski	red
22	11DC	Plavac	Sinj	red
23	12DC	Plavac	Šibenik	red
24	13DC	Plavac	Knin	red

**Table 2:** The investigated red and white wine samples from Croatian continental wine region of Hrvatsko zagorje.

Number	Sample Mark	Grape Variety	Origin	Type of Wine
1	1ZB	Graševina	Beretinec	white
2	2ZB	Pinot	Moslavec	white
3	3ZB	Rajnski rizling	Varaždin	white
4	4ZB	Graševina	Varaždin	white
5	5ZB	Muškat žuti	Varaždin	white
6	6ZB	Sivi pinot	Varaždin	white
7	7ZB	Manzoni	Beretinec	white
8	8ZB	Graševina	Varaždin	white
9	9ZB	Traminac	Beretinec	white
10	14ZB	Sauvignon	Cestica	white
11	1ZC	Frankovka	Beretinec	red
12	2ZC	Isabella + Farber	Kneginec	red
13	3ZC	Shiraz	Varaždin	red
14	4ZC	Frankovka + Plavac	Ludbreg	red
15	5ZC	Frankovka	Cestica	red
16	6ZC	Frankovka	Ledinec	red
17	7ZC	Merlot + Frankovka	Beretinec	red
18	8ZC	Portugizac	Ivanec	red
19	10ZC	Isabella	Ledinec	red
20	11ZC	Frankovka	Beretinec	red
21	12ZC	Frankovka + Merlot	Beretinec	red
22	13ZC	Isabella	Beretinec	red
23	14ZC	Isabella	Ledinec	red
24	15ZC	Frankovka	Beretinec	red

**Table 3:** The HPLC gradient elution program used for the analysis of biogenic amines.

Time (min)	Solvent A (%)	Solvent B (%)
0.5	40	60
25	80	20
30	95	5
34	95	5
35	40	60
43	40	60

**Table 4:** The analytical parameters of a chromatographic method.

Number	Amine Name (Short Name)	*t*_R_ (min) ± RSD (*n* = 3)	LOD (mg L^−1^)	LOQ (mg L^−1^)	*R* ^2^	Recovery White Wines (%)	Recovery Red Wines (%)	Linear Range (mg L^−1^)
1	Ethanolamine (ETHA)	5.79 ± 0.04	0.85	2.50	0.998	102	106	2.50–300.00
2	Methylamine (MA)	7.92 ± 0.05	0.14	0.41	0.999	123	88	0.41–100.00
3	Ethylamine (ETHYL)	9.07 ± 0.04	0.20	0.59	0.999	104	90	0.59–100.00
4	Tryptamine (TRP)	14.05 ± 0.04	0.03	0.06	0.998	103	82	0.06–215.00
5	Isopentylamine (IPA)	14.49 ± 0.04	0.22	0.66	0.999	107	93	0.66–200.00
6	Putrescine (PUT)	15.81 ± 0.05	0.03	0.09	0.998	97	88	0.09–96.70
7	Cadaverine (CAD)	16.71 ± 0.04	0.03	0.10	0.998	117	109	0.10–107.50
8	Histamine (HIS)	16.97 ± 0.04	0.03	0.10	0.998	99	91	0.10–107.50
9	Tyramine (TYR)	20.66 ± 0.04	0.04	0.13	0.999	107	92	0.13–107.50
10	Spermidine (SPD)	21.60 ± 0.03	0.03	0.09	0.998	107	101	0.09–96.70
11	Spermine (SPM)	26.12 ± 0.02	0.03	0.10	0.998	108	93	0.10–107.50

*t*_R_—retention time, RSD—relative standard deviation, LOD—limit of detection, LOQ—limit of quantification, *R*—correlation coefficient.

**Table 5:** The content of biogenic amines (mg L^−1^) in 48 investigated wine samples.

Sample	TRP	IPA	PUT	CAD	HIS	TYR	SPD	SPM	ETHA	MA	ETHYL
1DB	<0.03	<0.22	1.21 ± 0.04	<0.03	<0.03	<0.04	<0.03	<0.03	24.32 ± 8.05	<0.14	<0.59
2DB	<0.03	0.29 ± 0.21	1.19 ± 0.02	<0.03	<0.03	<0.04	<0.03	0.19 ± 0.09	10.42 ± 0.09	<0.14	<0.20
3DB	<0.03	<0.22	0.99 ± 0.04	<0.03	<0.10	0.39 ± 0.04	<0.03	0.39 ± 0.21	12.08 ± 0.83	1.40 ± 0.01	<0.20
4DB	0.49 ± 0.38	<0.22	0.41 ± 0.03	<0.03	<0.03	<0.04	<0.09	<0.10	9.09 ± 0.24	<0.14	<0.20
5DB	<0.03	<0.22	1.06 ± 0.00	<0.03	<0.03	<0.04	<0.09	<0.03	6.71 ± 0.00	<0.14	<0.20
6DB	0.42 ± 0.13	<0.22	0.86 ± 0.53	<0.03	<0.03	<0.04	<0.03	<0.03	2.87 ± 0.45	<0.14	<0.20
7DB	1.22 ± 1.12	<0.22	1.15 ± 0.01	<0.03	<0.03	<0.04	<0.03	0.11 ± 0.01	7.83 ± 0.33	<0.14	<0.20
8DB	0.68 ± 0.04	<0.22	1.25 ± 0.01	<0.03	0.56 ± 0.03	0.40 ± 0.01	<0.03	<0.10	4.22 ± 0.91	<0.14	<0.20
9DB	0.27 ± 0.18	<0.22	0.72 ± 0.02	<0.03	<0.03	<0.04	<0.03	<0.03	<2.5	<0.14	<0.20
10DB	0.38 ± 0.25	<0.22	2.10 ± 0.06	<0.10	<0.03	<0.04	<0.09	<0.03	<0.85	<0.14	<0.59
11DB	<0.03	<0.22	1.58 ± 0.02	<0.10	<0.03	<0.04	<0.03	<0.10	9.68 ± 0.04	<0.14	<0.59
1DC	<0.03	<0.22	1.58 ± 0.03	<0.03	<0.03	<0.04	<0.03	<0.03	4.85 ± 0.25	<0.14	<0.20
2DC	<0.03	1.13 ± 0.16	7.50 ± 0.17	<0.03	2.33 ± 0.08	2.00 ± 0.00	0.18 ± 0.04	0.31 ± 0.15	13.24 ± 0.13	<0.14	<0.20
3DC	0.54 ± 0.15	<0.22	1.76 ± 0.04	<0.10	0.89 ± 0.05	1.57 ± 0.01	<0.03	0.27 ± 0.13	14.00 ± 0.38	<0.14	<0.20
4DC	1.20 ± 0.01	<0.22	1.31 ± 0.00	<0.03	<0.03	<0.04	<0.03	<0.10	<0.85	<0.14	<0.20
5DC	0.43 ± 0.21	<0.22	1.35 ± 0.02	<0.03	<0.03	<0.04	0.09 ± 0.00	<0.03	<2.5	<0.14	<0.20
6DC	0.43 ± 0.18	<0.22	3.47 ± 0.09	<0.03	1.64 ± 0.01	1.88 ± 0.04	0.14 ± 0.05	0.24 ± 0.20	8.77 ± 0.50	<0.14	<0.20
7DC	0.23 ± 0.04	<0.22	0.78 ± 0.04	1.75 ± 0.03	0.89 ± 0.02	0.32 ± 0.03	<0.03	0.14 ± 0.03	5.11 ± 0.07	<0.14	<0.20
8DC	0.71 ± 0.04	<0.22	1.45 ± 0.02	<0.03	0.52 ± 0.01	<0.13	<0.03	<0.03	<2.5	<0.14	<0.20
9DC	0.34 ± 0.15	<0.22	0.78 ± 0.00	1.98 ± 0.09	1.17 ± 0.01	<0.13	0.46 ± 0.25	<0.10	9.22 ± 0.06	<0.14	<0.20
10DC	0.43 ± 0.35	<0.22	1.91 ± 0.11	<0.03	<0.03	<0.04	<0.03	0.17 ± 0.08	<0.85	<0.14	<0.20
11DC	<0.03	<0.22	1.77 ± 0.01	0.12 ± 0.06	<0.03	<0.04	0.12 ± 0.01	<0.03	10.75 ± 0.45	<0.14	<0.20
12DC	0.46 ± 0.30	<0.22	2.77 ± 0.07	<0.03	<0.03	<0.13	<0.09	<0.10	6.58 ± 0.17	<0.14	<0.20
13DC	0.66 ± 0.04	<0.22	0.89 ± 0.03	1.01 ± 0.14	0.36 ± 0.04	<0.04	<0.03	<0.03	6.36 ± 0.03	<0.14	<0.59
1ZB	0.68 ± 0.02	1.29 ± 0.04	0.27 ± 0.01	0.40 ± 0.00	<0.03	<0.04	<0.03	<0.10	4.07 ± 0.28	<0.14	<0.20
2ZB	<0.03	0.87 ± 0.49	1.49 ± 0.67	0.82 ± 0.31	<0.03	<0.04	<0.03	<0.03	17.96 ± 0.01	<0.14	<0.20
3ZB	<0.03	1.32 ± 0.00	0.62 ± 0.01	<0.03	<0.03	<0.04	<0.03	<0.03	4.80 ± 0.51	<0.14	<0.20
4ZB	<0.03	1.47 ± 0.32	1.19 ± 0.01	<0.03	<0.03	<0.04	<0.03	<0.10	8.61 ± 1.19	<0.14	<0.20
5ZB	<0.03	0.50 ± 0.09	1.08 ± 0.01	<0.03	<0.03	<0.04	<0.03	<0.03	<0.85	<0.14	<0.20
6ZB	<0.03	0.82 ± 0.21	<0.09	<0.03	<0.03	<0.04	<0.03	<0.03	3.80 ± 0.00	<0.14	<0.20
7ZB	<0.03	0.42 ± 0.46	1.42 ± 0.01	<0.03	<0.03	<0.04	<0.03	<0.03	4.61 ± 0.01	<0.14	<0.20
8ZB	<0.03	0.31 ± 0.22	0.83 ± 0.04	<0.03	<0.03	<0.04	0.12 ± 0.02	<0.10	4.16 ± 0.14	<0.14	<0.20
9ZB	0.33 ± 0.00	1.00 ± 0.80	1.16 ± 0.02	<0.03	<0.10	<0.04	<0.03	<0.10	7.19 ± 0.08	<0.14	<0.20
14ZB	<0.03	<0.22	0.94 ± 0.01	<0.10	<0.03	<0.04	<0.09	<0.03	<0.85	<0.14	<0.59
1ZC	0.75 ± 0.87	<0.22	0.17 ± 0.01	<0.03	<0.03	<0.13	0.21 ± 0.05	<0.03	<2.5	<0.14	<0.20
2ZC	1.82 ± 0.06	0.79 ± 0.32	2.06 ± 0.04	0.47 ± 0.05	1.06 ± 0.04	<0.13	0.75 ± 0.04	3.55 ± 0.04	15.41 ± 0.02	<0.14	<0.59
3ZC	3.31 ± 0.33	1.19 ± 0.01	3.75 ± 0.06	<0.10	9.63 ± 0.13	<0.13	0.26 ± 0.13	0.74 ± 0.04	4.10 ± 0.23	1.28 ± 0.01	<0.59
4ZC	4.89 ± 0.27	0.50 ± 0.06	0.39 ± 0.04	<0.10	0.35 ± 0.08	0.45 ± 0.01	0.15 ± 0.01	0.67 ± 0.07	8.04 ± 0.16	<0.14	<0.20
5ZC	3.61 ± 0.45	0.87 ± 0.04	3.17 ± 0.11	0.10 ± 0.00	0.50 ± 0.01	0.50 ± 0.04	0.22 ± 0.02	1.81 ± 0.01	16.80 ± 0.23	1.57 ± 0.04	<0.59
6ZC	0.88 ± 0.22	3.71 ± 0.14	<0.03	<0.10	<0.03	0.31 ± 0.08	2.51 ± 0.21	<0.03	95.84 ± 0.20	0.99 ± 0.55	<0.20
7ZC	0.39 ± 0.31	2.44 ± 0.18	0.66 ± 0.78	0.21 ± 0.01	0.98 ± 0.02	2.10 ± 0.14	0.57 ± 0.07	<0.03	63.96 ± 0.20	<0.14	<0.20
8ZC	0.09 ± 0.01	2.97 ± 2.44	<0.09	<0.03	<0.10	0.78 ± 0.71	0.33 ± 0.00	<0.03	31.14 ± 0.17	0.65 ± 0.03	<0.20
10ZC	0.67 ± 0.37	1.81 ± 0.02	0.16 ± 0.09	0.44 ± 0.20	<0.03	2.31 ± 0.05	0.15 ± 0.00	<0.03	51.79 ± 3.21	<0.14	<0.20
11ZC	9.18 ± 0.07	2.14 ± 0.48	<0.09	<0.10	<0.03	1.45 ± 0.22	6.05 ± 1.22	<0.03	35.19 ± 10.47	0.56 ± 0.15	<0.59
12ZC	0.87 ± 0.61	3.27 ± 0.35	<0.09	0.27 ± 0.32	2.01 ± 0.14	2.97 ± 0.09	1.97 ± 0.20	<0.03	46.03 ± 12.33	<0.41	1.17 ± 0.21
13ZC	<0.03	1.55 ± 1.27	1.00 ± 0.04	<0.03	0.35 ± 0.06	0.13 ± 0.03	<0.03	<0.10	23.78 ± 1.50	<0.14	<0.20
14ZC	<0.03	0.33 ± 0.32	0.24 ± 0.02	<0.10	<0.03	<0.04	<0.09	<0.10	5.22 ± 0.28	<0.14	<0.20
15ZC	<0.03	0.70 ± 0.10	<0.03	<0.03	<0.03	<0.04	<0.03	<0.10	4.37 ± 0.20	<0.14	<0.20

The table contains abbreviations for biogenic amines, and the corresponding full names are reported in Table 4. DB = Dalmatian white samples, DC = Dalmatian red samples, ZB = Hrvatsko zagorje white samples and ZC = Hrvatsko zagorje red samples.

**Table 6:** Loading values for PCA.

	PC1	PC2	PC3	PC4
TRP	**0.3160**	0.2615	−0.4331	0.1254
IPA	**0.4612**	−0.1534	0.0624	−0.1102
PUT	−0.0565	**0.4970**	0.3967	−0.1822
CAD	−0.0167	−0.0588	0.1155	0.9085
HIS	0.1533	**0.4641**	0.3395	0.0760
TYR	**0.3744**	−0.0824	0.4237	−0.0500
SPD	**0.4325**	−0.0409	−0.3308	0.0847
SPM	**0.3160**	0.2615	−0.4331	0.1254
ETHA	**0.4612**	−0.1534	0.0624	−0.1102
MA	−0.0565	**0.4970**	0.3967	−0.1822
ETHYL	−0.0167	−0.0588	0.1155	0.9085

Short amine names correspond to amines’ name in Table 4.

**Table 7:** Explanation of variance in statistical analysis.

	PC1	PC2	PC3	PC4
Standard deviation	1.8279	1.4114	1.1870	1.03047
Proportion of variance	0.3038	0.1811	0.1281	0.09653
Cumulative proportion	0.3038	0.4849	0.6130	0.70949
